# Internal Iliac Artery Stenosis: Diagnosis and How to Manage it in 2015

**DOI:** 10.3389/fcvm.2015.00033

**Published:** 2015-09-01

**Authors:** Guillaume Mahé, Adrien Kaladji, Alexis Le Faucheur, Vincent Jaquinandi

**Affiliations:** ^1^Pôle Imagerie et Explorations Fonctionnelles, Rennes, France; ^2^INSERM, Clinical Investigation Center (CIC 14 14), Rennes, France; ^3^Vascular Surgery, University Hospital, Rennes, France; ^4^INSERM Laboratoire Traitement du Signal et de l’Image (LTSI), UMR 1099, Rennes, France; ^5^École normale supérieure de Rennes, Rennes, France; ^6^Vascular Medicine, Trélazé, France

**Keywords:** internal iliac artery stenosis, management, methods, surgery, peripheral artery disease

## Abstract

Lower extremity arterial disease (LEAD) is a highly prevalent disease affecting 202 million people worldwide. Internal iliac artery stenosis (IIAS) is one of the localization of LEAD. This diagnosis is often neglected when a patient has a proximal walking pain since most physicians evoke a pseudoclaudication. Surprisingly, IIAS management is reported neither in the Trans-Atlantic Inter-Society Consensus II nor in the report of the American College Foundation/American Heart Association guidelines. The aims of this review are to present the current knowledge about the disease, how should it be managed in 2015 and what are the future research trends.

Lower extremity arterial disease (LEAD) is a highly prevalent disease mainly caused by atherosclerosis, a systemic disease process that alters the normal structure and function of the vessels (1). Thus, LEAD risk factors are well identified: non-modifiable risk factors such as age, gender, and heredity; and modifiable risk factors such as smoking, hypertension, diabetes, and dyslipidemia (2).

It is common to define proximal LEAD and distal LEAD depending on the ischemia area supplied by the damage artery (3). Twenty to 50% LEAD patients are asymptomatic (4). When the claudication is present, the distal LEAD is typically characterized by calf pain and relies on either common iliac lesion and/or external iliac artery and/or femoropopliteal lesions (2). Contrary to distal LEAD, the proximal LEAD is characterized by lower back, hip, buttock, or thigh pain and relies on either common iliac and/or isolated internal iliac lesions (2, 3, 5, 6).

Internal iliac artery stenosis (IIAS) is one of the possible localizations of atherosclerosis on the arterial tree. This disease is often missed in the diagnosis process when a patient has a proximal walking pain. Indeed, a pain that appears during walking and involves the lower back, hip, buttock, or thigh suggests either proximal claudication or proximal pseudoclaudication (2). Claudication is a vasculogenic pain whereas pseudoclaudication results from diseases such as lumbar spinal stenosis, hip osteoarthritis, venous congestion, or bone metastasis, sciatica, and so on (7–9) (Figure 1; Table 1).

**Figure 1:**
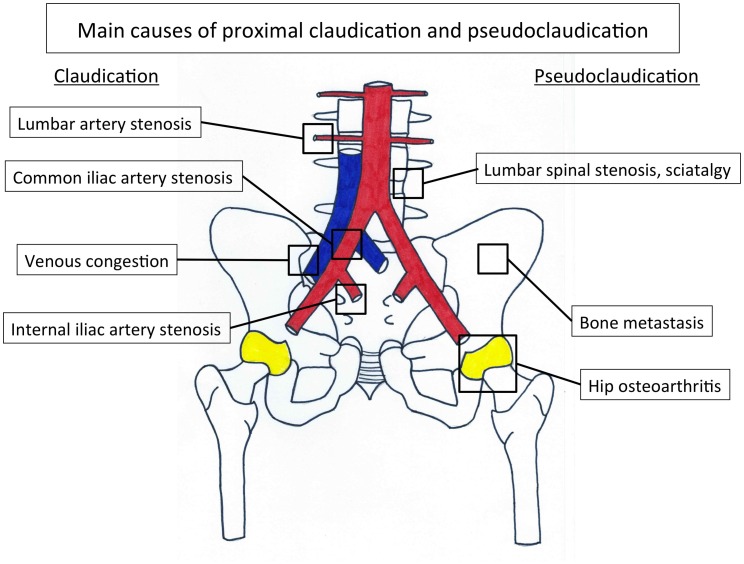
**Main causes of proximal claudication and pseudoclaudication**.

**Table 1:** **Differential diagnosis of internal iliac artery stenosis**.

Potential disease	Location of pain or discomfort	Characteristic discomfort	Exercise-induced discomfort	Effect of rest	Effect of body position	Other characteristics
Arterial atherosclerotic disease (intermittent claudication)	Buttock, hip, lower back, thigh	Cramping, aching, fatigue, weakness, or frank pain	Yes	Rapid relief with rest	None	Presence of cardiovascular risk factors
Spinal stenosis	Buttock, hip, thigh	Cramping, aching, fatigue, weakness or tingling or clumsiness	Variable	Relieved by sitting or changing position	Relief by lumbar spine flexion (sitting or stooping forward)	History of back problems
Hip osteoarthritis	Buttock, hip, thigh	Aching	Variable	Not rapid relief (and may be present at rest)	More comfortable sitting	Related to activity level, weather changes
Bone metastasis	At the bone level	Aching	Variable	Not rapid relief (and may be present at rest)	Avoid direct pressure on bones	History of cancer
Venous congestion	Thigh, groin	Tightness, bursting	After walking	Subsides slowly	Relief by elevation	History of deep veins thrombosis at the inferior cava or iliac level; presence of varicoses

*Adapted from Hirsh et al. (2) and White C (9)*.

Unfortunately, due to nearly similar symptoms, the vascular origin is largely neglected when a patient has a proximal limb pain since most physicians evoke a pseudoclaudication (9).

Several explanations can be proposed. First, claudication was historically defined by a fatigue, discomfort, or pain occurring in calves during effort due to exercise-induced ischemia and which is relieved with rest (10). Second, ankle-brachial index (ABI) is used as the standard for the diagnosis of LEAD (11). Indeed, LEAD is defined by an ABI ≤0.90 (11), but the latter can remain within normal limit in case of isolated IIAS (12) or superior gluteal artery lesions (13). Finally, IIAS management is reported neither in the Trans-Atlantic Inter-Society Consensus II (TASC II) nor in the report of the American College Foundation/American Heart Association (AHA) guidelines (4, 11).

## Incidence and Prevalence of IIAS

Incidence and prevalence of IIAS have not been established in general population. Although isolated IIAS is probably rare, IIAS is often associated with common iliac artery stenosis. The prevalence of proximal claudication is 5–14% in patients with mild-to-moderate distal LEAD (5, 14), appears high among patients with patent aortobifemoral bypasses (15), approximately 28%, and nearly 35% of patients after bilateral internal iliac artery (IIA) embolization prior to endovascular aneurysm repair (16).

## Symptoms and Functional Impairment

The main symptom is the lower back, hip, buttock, or thigh claudication defining the proximal claudication, a fatigue, discomfort, or pain occurring in specific muscle groups alimenting by IIA during effort due to exercise-induced ischemia and which is relieved with rest (2, 6). However, the presentation of proximal claudication is often atypical and may mimic other non-vascular diseases that may induce misleading diagnoses (5, 9) (Figure 1). Moreover, the pain may appear at rest when IIAS is severe, and can lead even to gluteal necrosis (17). Finally, IIAS induces different functional impairments: walking impairment that leads to work disability, and sexual impairment as erectile dysfunction (16, 18). These two impairments reduce the patient quality of life (19–21). To avoid these impairments, although it is challenging with conventional non-invasive tests, it is of interest to diagnose the disease in order to diminish the delay of diagnosis, which is currently of 2 years, when the disease is not associated with distal LEAD (14).

## Strategies and Evidences

### Clinical evaluation

Patients typically report a gradual decrease of their walking capacity, which is evaluated by the maximal walking distance (2). Claudication traditionally stops within 10 min after the end of walking (22, 23). The maximal walking distance often impairs either with the increase of the arterial stenosis or the deterioration of the functionality of the collateral network, which both diminish blood flow delivery to muscles irrigated by the IIA (6). The maximal walking distance can be evaluated by different methods: medical interview, questionnaires (24), standardized treadmill evaluation which is recommended (11), 6-min test when patients are unable to walk on a treadmill (11), and recently GPS technology (25).

Physical examination should rule out a pseudoclaudication (9). Hip osteoarthritis is associated with pain typically in the groin provoked by internal or external rotation of the hip (8). The hip pain often radiates in the knee, whereas lumbar spinal stenosis is associated with discomfort that radiates beyond the spinal area into the buttocks (7). Furthermore, active lumbar extension may induce discomfort that is relieved with flexion. Finally, pain due to bone metastasis is aggravated by direct pressure on bones.

Different questionnaires have been developed to help clinicians to distinguish vascular claudication from pseudoclaudication. The most used are the World Health Organization (WHO)/Rose Questionnaire, the Edinburgh claudication questionnaire, and the San Diego questionnaire (22, 26, 27) [Readers will find these entire questionnaires in the same article published in Vascular Medicine (28)].

Pulse palpations, auscultation of lower limbs, and abdomen looking for a vascular breath have to be done on patient (2).

The ABI measurement defined as the highest systolic pressure in the dorsalis pedis or posterior tibial artery divided by the highest arm pressure measurement can be helpful, although it may be insensitive in the case of isolated internal iliac lesions (11, 12).

The penile pressure measurement has been suggested to be an alternative method to diagnose proximal arteries stenosis (29). Penile-brachial index (PBI), defined as penile pressure over the highest systolic brachial ratio, is rarely used in routine investigations, and its accuracy is 69.3% (95% confidence interval: 58.6–78.7) for the detection of an arterial stenosis or occlusion on at least one side as compared with the arteriograms (30). Therefore, a normal PBI (>0.60) cannot rule out the presence of lesions on the internal iliac arteries (30).

#### Classical Imaging

Duplex ultrasound (DUS) of the vascular abdominal circulation may help to evoke the diagnosis (11) (Table 2). Indeed, the sensitivity and specificity of DUS to assess aorto-iliac disease (stenoses) when compared with arteriography are 91% and 93%, respectively (31). However, no study has evaluated the sensitivity and specificity of DUS in the specific assessment of IIAS. Sensitivity and specificity obtained for the aorto-iliac disease are probably higher than those expected in the assessment of IIAS because the IIA is deeper and more difficult to evaluate with DUS. Some authors have also suggested assessing indirectly the IIA by studying the gluteal artery with DUS but the results have to be confirmed on larger population (32).

**Table 2:** **Different tests that can be used to diagnose the internal iliac artery stenosis**.

	Non-invasive test							Invasive test
	ABI/Post-exercise ABI	Continuous-wave Doppler waveforms	Penile-brachial index	DUS	CTA/MRA	Exercise-NIRS	Exercise-TcPO_2_	Digital subtraction angiography
Arteries assessed (direct or indirect assessment)								
Aorta	Yes/Indirect	Yes/Direct	Yes/Indirect	Yes/Direct	Yes/Direct	Yes/Indirect	Yes/Indirect	Yes/Direct
Common Iliac artery	Yes/Indirect	Yes/Direct	Yes/Indirect	Yes/Direct	Yes/Direct	Yes/Indirect	Yes/Indirect	Yes/Direct
Internal iliac artery	No	No	Yes/Indirect	Yes/Direct	Yes/Direct	Yes/Indirect	Yes/Indirect	Yes/Direct
External iliac artery	Yes/Indirect	Yes/Direct	No	Yes/Direct	Yes/Direct	Yes/Indirect	Yes/Indirect	Yes/Direct
Limb artery	Yes/Indirect	Yes/Direct	No	Yes/Direct	Yes/Direct	Yes/Indirect	Yes/Indirect	Yes/Direct
Type of assessment	Functional	Functional	Functional	Functional and morphological	Morphological	Functional	Functional	Morphological
Patient’s condition	at rest	at rest	at rest	at rest	at rest	during exercise	during exercise	at rest
Availability	Every center	Every center	Specialized center	Every center	Every center	Specialized center	Specialized center	Every center
Cost	+	+	+	++	+++	++	++	+++

*ABI, ankle-brachial index; DUS, duplex ultrasound; CTA, computerized-tomodensitometric angiography; MRA, magnetic-resonance angiography (MRA); Exercise-TcPO_2_, exercise transcutaneous oxygen pressure; NIRS, exercise near-infrared spectroscopy*.

**Figure 2:**
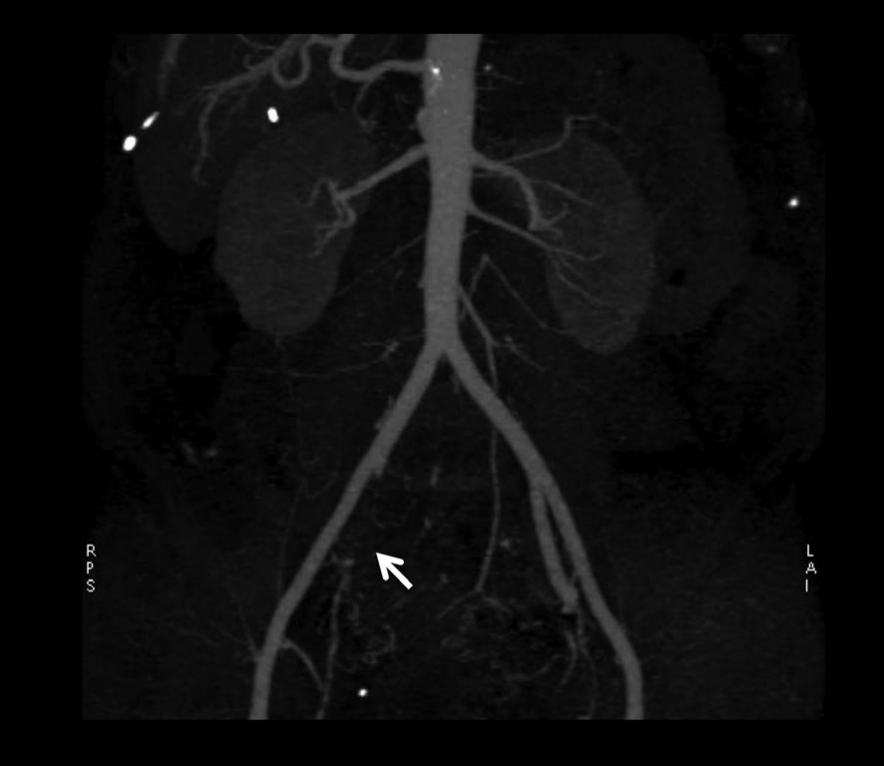
**Results of computerized-tomodensitometric angiography: occlusion of the right internal iliac artery**. Arrow, occlusion of the right internal iliac artery.

Computerized-tomodensitometric angiography (CTA) and magnetic-resonance angiography (MRA), “semi-invasive” exams, can be an option for the diagnosis of IIAS especially when a patient is candidate for revascularization (Figure 2) (11).

Digital subtraction angiography, an invasive technique remains the gold standard for the diagnosis of IIAS with oblique projections to avoid missing the diagnosis of IIAS (11). Indeed, the external iliac artery hides the IIA at the level of gluteal canal on antero-posterior views (33). Moreover, as suggested by the American College of Cardiology (ACC) and the AHA, when conducting a diagnostic lower extremity arteriogram in which the significance of an obstructive lesion is ambiguous, transstenotic pressure gradients should be obtained (2). The cut-off of mean resting gradient to determine whether or not the stenosis is significant is discussed ranging from 5 (34) to 10 mm Hg (35). The peripheral fractional flow reserve (p-FFR), which is defined as the ratio of the mean pressure distal to the stenosis to the mean aortic pressure at hyperemia, could also be a valuable test (36–38).

#### Functional Assessment of IIAS during Walking

**Figure 3:**
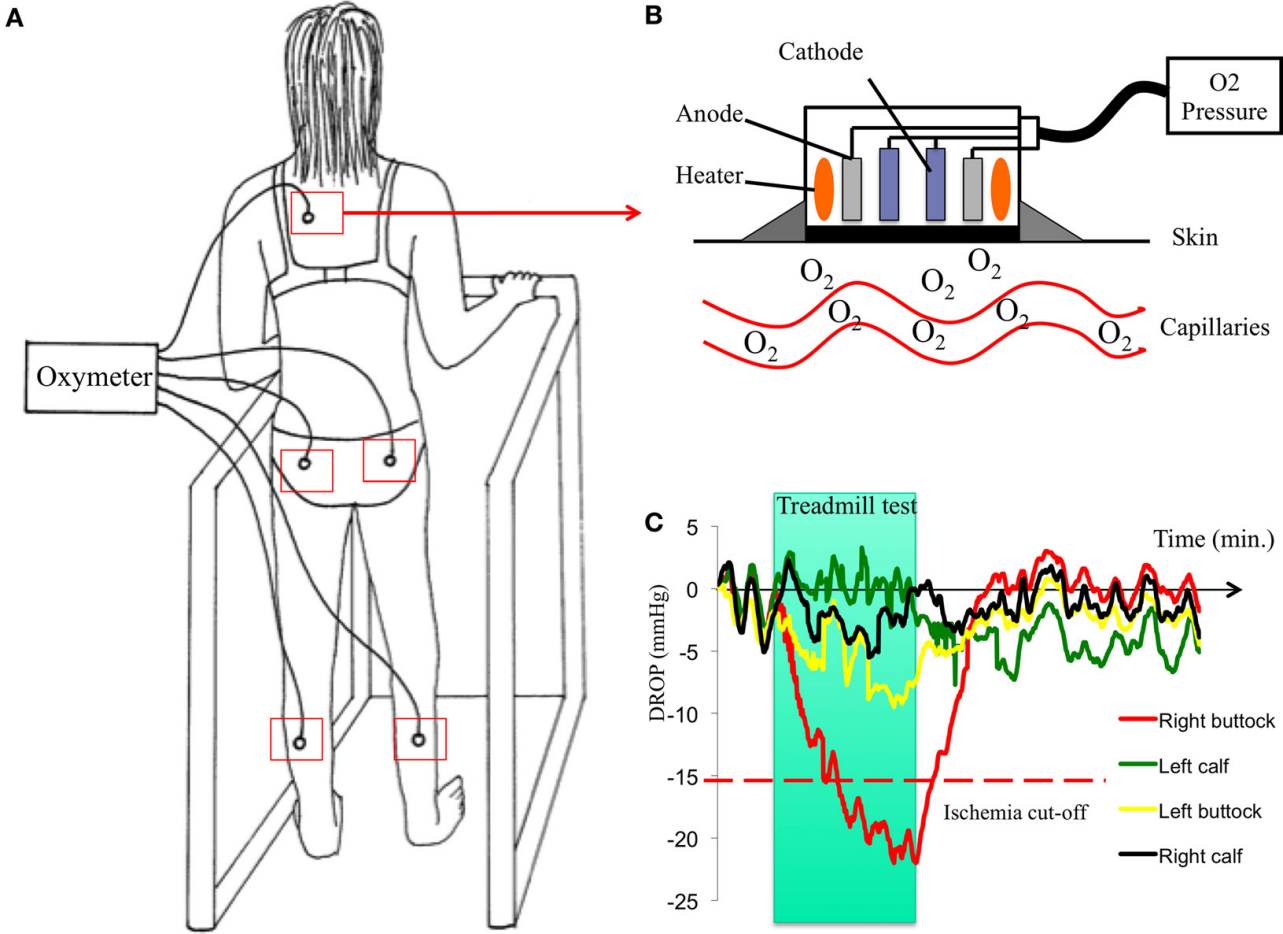
**Procedure of Exercise-TcPO_2_ in one patient**. Case history: a 59-year-old woman, former smoker with a history of hypertension and hyperlipidemia always reports pain in her right buttock when she walks although she had surgery for lumbar spinal stenosis 2 years before. The discomfort has progressively worsened over the past 4 months and now forces her to rest after walking 250 m at a normal pace. The pain is interfering with her ability to perform her job. She has a normal right femoral pulse and normal ankle-brachial index. **(A)**: patient on a treadmill with five probes of TcPO_2_ (one on each buttock, one on each calf, and one on the chest) and a 12-lead electrocardiogram. Upper right **(B)**: schema of a TcPO_2_ probe. Lower right **(C)**: typical recordings of Exercise-TcPO_2_ measurements showing a right buttock ischemia with a DROP (delta from rest of oxygen pressure) lower than −15 mm Hg.

Both exercise transcutaneous oxygen pressure (Exercise-TcPO_2_) and exercise near-infrared spectroscopy (NIRS) measurements performed on treadmill have been proposed to estimate exercise-induced ischemia at the buttock level (3, 37, 39, 40). Exercise-TcPO_2_ reflects skin oxygenation whereas NIRS reflects muscles oxygenation. Nevertheless, at the buttock level at least, the skin and muscles below are vascularized by the same arterial trunk, the internal artery. Compared in the same study, NIRS and Exercise-TcPO_2_ provided, respectively, 55% (range, 41.6–67.9%) and 82% (range, 69.6–90.5%) accuracy (95% confidence interval) to predict the presence of arteriographically proven lesions (41). The Exercise-TcPO_2_ has 79% sensibility and 86% specificity to detect significant lesions (stenosis ≥75%) in the arterial tree of the pelvic circulation when the cut-off point is lower then −15 mm Hg (3) (Figure 3). The Exercise-NIRS provides a non-invasive method of measuring tissue oxygen saturation [StO(2)]. Different parameters have been proposed using different treadmill protocols (39, 42, 43). Time to 50% of StO(2) recovery to baseline [T(50)] >70 s yielded a sensitivity of 89% and a specificity of 85% for PAD (42). To predict a significant lesion on the arteries toward the hypogastric circulation, a recovery time >240 s was considered as a cut-off value (39).

#### Management

There is no specific management of IIAS (4, 11). The medical management is the same as for LEAD. To reduce adverse cardiovascular events such as stroke and acute myocardial infarction, lifelong therapy should include elimination or modification of atherosclerotic modifiable risk factors such as smoking, hypertension, diabetes mellitus, and dyslipidemia (11). Daily exercise and favorable diet limiting the atherosclerotic process are recommended (9, 11). Revascularization indication depends on the patient functional impairment (e.g., normal work or other activities important for the patient) after a lack of adequate response to exercise therapy and well-conducted medical treatment (11). The morphology of the lesion is also an important criterion for the choice of the revascularization (4, 11).

#### Lifestyle Intervention and Atherosclerotic Risk Factors Modifications

Smoking cessation is needed because continuing smoking compared with smoking cessation was found to increase the risk of death, myocardial infarction, and amputation (44). Patients who are smokers or former smokers should be asked about tobacco use status at every visit and be assisted to stop (11). The WHO study group has proposed that guidelines for the prevention of cardiovascular disease include a diet low in fat (<30% of calories) and saturated fatty acids (<10% of calories) and devoid of trans fatty (saturated) acids. Salt intake should be reduced to 5 g/d (90 mmol/d). Consumption of a broad range of fruits, vegetables (>400 g/d corresponding to five intakes per day) is encouraged (45). Thus, dietary assessment has to be done in clinical practice with specific validated tool (46).

Patients with proximal claudication could benefit from home-based exercise program or supervised exercise training with at least three sessions of 35 to 45 min per week for a minimum of 12 weeks (11, 47). Therefore, physical activity has to be highly recommended (45).

### Medical treatment

In addition to lifestyle changes, antiplatelet agents as aspirin in daily doses of 75–325 mg is recommended as safe and effective therapy to reduce the risk of myocardial infarction, stroke, and vascular death (48). Clopidogrel (75 mg per day) can be an alternative antiplatelet therapy since it appears to be more effective than aspirin in preventing ischemic events in patients with symptomatic LEAD without increasing bleeding events (CAPRIE study) (49).

Lipid-lowering drugs are also recommended for all LEAD patients to achieve a goal of LDL cholesterol level less than 100 mg per dL, and when risk is very high, a LDL cholesterol target of less than 70 mg/dL is preferable (11). However, the ACC/AHA guidelines published in 2013 have stopped to recommend a cholesterol target since clinical trial data do not indicate what the target should be. According to these guidelines, in case of atherosclerotic cardiovascular disease, a high intensity statin should be used for patients aged ≤75 years old or a moderate intensity statin for patients aged >75 years old (50). In the Heart Protection Study trial, simvastatin (a hydroxymethyl glutaryl (HMG) coenzyme-A reductase) has significantly reduced the risk of major vascular event by 22% (95% confidence interval: 15–29) in LEAD patients as compared to placebo (51). Antihypertensive drugs should be administered to LEAD patients with hypertension to achieve a goal of <140 mm Hg systolic over 90 mm Hg diastolic (in non-diabetic patients) or <130 mm Hg systolic over 80 mm Hg diastolic (diabetic patients and patients with chronic renal disease) (11). The angiotensin-converting enzyme inhibitors (ACEI) should be preferred in asymptomatic or symptomatic LEAD to reduce the risk of adverse cardiovascular events since ramipril 10 mg/day diminished the risk of myocardial infarction, stroke, or vascular death in patients with LEAD by approximately 25% (52). Ramipril resulted also in significant increases in pain-free and maximum treadmill walking times compared with placebo (53). In practice, the introduction of ACEI should be done over 1 month by increasing the dose slowly and monitoring the creatinine clearance.

In diabetic patients, antidiabetic drugs should be administered to reach a goal of Hemoglobin A1c below 7% to reduce microvascular complications and improve cardiovascular ­outcomes (11).

Others drugs as cilostazol or pentoxifylline can be used in patients with LEAD to improve the walking distance (11).

### Revascularization

Revascularization can be proposed to patients who have an important walking impairment and after no improvement after medical treatment and supervised exercise training (4, 11). In case of isolated IIAS, endovascular treatment is the most used procedure since surgical revascularization is both technically more demanding and brings along a higher risk to the patient (54, 55). Contrary to stenosis concerning the common iliac artery or external iliac artery, there is neither randomized comparison of primary stent placement versus percutaneous transluminal angioplasty (PTA) nor comparison with surgery for IIAS (35, 56). Several studies have assessed the endovascular treatment (PTA alone or stenting) on short series of patients. Thompson et al. have shown that the procedures either for PTA alone or stenting were safe without any complication in nine patients with buttock claudication: seven out of nine had pain relief after 1 month of follow-up (54). Another study, including 21 patients followed during 14.7 ± 5.7 months, found that all patients had complete alleviation of the buttock claudication and a significant increase of the walking distance from 85 m to 225 m after the endovascular treatment (PTA alone or stenting) (55). In 2013, Prince et al. have shown that endovascular treatment of IIAS has a high technical success rate (i.e., absence or stenosis after procedure lower than 30%) and a low complication rate (in 3 out of 34 patients). In their study (34 patients included), 79% of patients had complete or partial pain relief of symptoms (57). Morse et al., Smith et al., Adlakha et al., and Huetink et al. have also reported different symptomatic cases of IIAS that were successfully treated by endovascular procedures (58–61). Batt et al. have also shown good results for the angioplasty of the superior gluteal artery lesions (13, 62). Paumier et al. have suggested that in case of common iliac artery stenosis, aorto-iliac bypasses with a reimplantation of IIA should be discussed (63). The same group performed another study in 40 patients, in whom a direct revascularization of the IIA was performed concomitant to aorto-or iliofemoral bypasses. Proximal claudication after revascularization disappeared in 23 out 27 patients who had previous proximal claudication (64). The patency rate of the IIA was 89% at 1 year and 72.5% at 5 years (64).

## Areas of Uncertainties

### Algorithm of diagnosis

**Figure 4:**
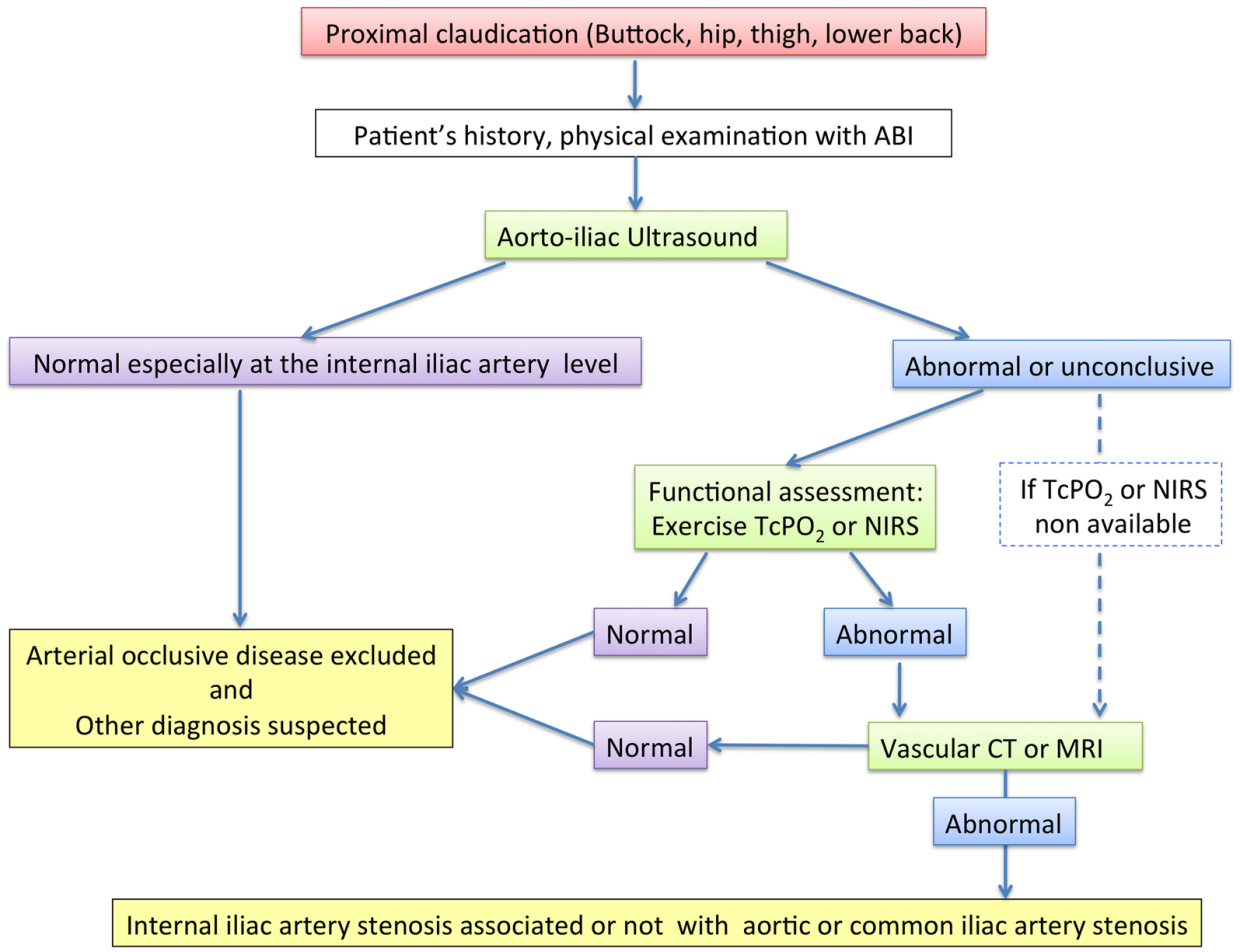
**Diagnosis algorithm**. ABI, ankle-brachial index; Exercise-TcPO_2_, exercise transcutaneous oxygen pressure measurement; Exercise-NIRS, near-infrared spectroscopy; CT, computed tomography.

To date, no algorithm has been proposed to perform the IIAS diagnosis taking account the cost of the exams and their possible results to help for the diagnosis. Studies are lacking about the sensitivity and specificity of several exams such as ultrasound exam, CTA, or MRA on the specific IIAS evaluation. Herein, we propose our diagnosis algorithm (Figure 4).

### Percutaneous transluminal angioplasty alone or stenting

Randomized comparison of primary stent placement versus angioplasty concerning common iliac artery or external iliac artery had shown that there were no substantial differences in technical results and clinical outcomes of the two treatment strategies both at short-term and long-term follow-up (2 years) (35). Since angioplasty followed by selective stent placement is less expensive than direct placement of a stent, the choice of primary stenting or not has to be studied for the IIAS.

### Assessment of other atherosclerotic localizations

An electrocardiogram, carotid, and aortic DUS should be discussed because LEAD is associated half the time with other atherosclerotic localizations (65).

## Guidelines

An expert multidisciplinary committee organized by the ACC and the AHA have reported comprehensive guidelines for the management of LEAD in 2013 (11). Surprisingly, neither this committee nor the TASC II working group mentioned the way of managing IIAS (4, 11). In 2014, the Society for Cardiovascular Angiography and Intervention (SCAI) has published an expert consensus statement about aorto-iliac arterial management (34). In this statement, the experts considered as:

- appropriate care to treat a patient with moderate to severe symptoms of buttock of hip claudication or major tissue loss (Rutherford Classification: 2–6) with IIA ≥50% stenosis and/or resting mean translesional gradient ≥5 mm Hg;
- may be appropriate care to treat a patient with IIAS ≥50% with vasculogenic impotence.

## Conclusion

This review shows that when a patient has a proximal walking pain, physician should look for IIAS. We also found that literature is very sparse and that guidelines and recommendations have to be addressed to know how to manage IIAS.

## Conflict of Interest Statement

The authors declare that the research was conducted in the absence of any commercial or financial relationships that could be construed as a potential conflict of interest.

## References

[1] Fowkes FG Rudan D Rudan I Aboyans V Denenberg JO McDermott MM Comparison of global estimates of prevalence and risk factors for peripheral artery disease in 2000 and 2010: a systematic review and analysis. Lancet (2013) 382(9901):1329–40. 10.1016/S0140-6736(13)61249-0 23915883

[2] Hirsch AT Haskal ZJ Hertzer NR Bakal CW Creager MA Halperin JL ACC/AHA 2005 practice guidelines for the management of patients with peripheral arterial disease (lower extremity, renal, mesenteric, and abdominal aortic): a collaborative report from the American association for vascular surgery/society for vascular surgery, society for cardiovascular angiography and interventions, society for vascular medicine and biology, society of interventional radiology, and the ACC/AHA task force on practice guidelines (writing committee to develop guidelines for the management of patients with peripheral arterial disease): endorsed by the American association of cardiovascular and pulmonary rehabilitation; national heart, lung, and blood institute; society for vascular nursing; transatlantic inter-society consensus; and vascular disease foundation. Circulation (2006) 113:e463–654. 10.1161/CIRCULATIONAHA.106.174526 16549646

[3] Abraham P Picquet J Vielle B Sigaudo-Roussel D Paisant-Thouveny F Enon B Transcutaneous oxygen pressure measurements on the buttocks during exercise to detect proximal arterial ischemia: comparison with arteriography. Circulation (2003) 107:1896–900. 10.1161/01.CIR.0000060500.60646.E0 12668524

[4] Norgren L Hiatt WR Dormandy JA Nehler MR Harris KA Fowkes FG Inter-society consensus for the management of peripheral arterial disease (TASC II). J Vasc Surg (2007) 45:S5–67. 10.1016/j.jvs.2006.12.037 17223489

[5] Jaquinandi V Bouye P Picquet J Leftheriotis G Saumet JL Abraham P. Pain description in patients with isolated proximal (without distal) exercise-related lower limb arterial ischemia. Vasc Med (2004) 9:261–5. 10.1191/1358863x04vm560oa 15678617

[6] Jaquinandi V Abraham P Picquet J Paisant-Thouveny F Leftheriotis G Saumet JL. Estimation of the functional role of arterial pathways to the buttock circulation during treadmill walking in patients with claudication. J Appl Physiol (2007) 102:1105–12. 10.1152/japplphysiol.00912.2006 17110509

[7] Katz JN Harris MB Clinical practice. Lumbar spinal stenosis. N Engl J Med (2008) 358:818–25. 10.1056/NEJMcp0708097 18287604

[8] Lane NE Clinical practice. Osteoarthritis of the hip. N Engl J Med (2007) 357:1413–21. 10.1056/NEJMcp071112 17914042

[9] White C Clinical practice. Intermittent claudication. N Engl J Med (2007) 356:1241–50. 10.1056/NEJMcp064483 17377162

[10] Bouley JF Claudication intermittente des membres postérieurs, déterminée par l’oblitération des artères fémorales. Recueil Méd Vét (1831) 8:517–27.

[11] Rooke TW Hirsch AT Misra S Sidawy AN Beckman JA Findeiss L Management of patients with peripheral artery disease (compilation of 2005 and 2011 ACCF/AHA guideline recommendations): a report of the American college of cardiology foundation/American heart association task force on practice guidelines. J Am Coll Cardiol (2013) 61:1555–70. 10.1016/j.jacc.2013.01.004 23473760 PMC4492473

[12] Gernigon M Marchand J Ouedraogo N Leftheriotis G Piquet JM Abraham P. Proximal ischemia is a frequent cause of exercise-induced pain in patients with a normal ankle to brachial index at rest. Pain Physician (2013) 16:57–64. 23340534

[13] Batt M Baque J Ajmia F Cavalier M. Angioplasty of the superior gluteal artery in 34 patients with buttock claudication. J Endovasc Ther (2014) 21:400–6. 10.1583/13-4676R.1 24915588

[14] Picquet J Jaquinandi V Saumet JL Leftheriotis G Enon B Abraham P. Systematic diagnostic approach to proximal-without-distal claudication in a vascular population. Eur J Intern Med (2005) 16:575–9. 10.1016/j.ejim.2005.06.013 16314239

[15] Jaquinandi V Picquet J Bouyé P Saumet JL Leftheriotis G Abraham P High prevalence of proximal claudication among patients with patent aortobifemoral bypasses. J Vasc Surg (2007) 45:318–8. 10.1016/j.jvs.2006.09.050 17264010

[16] Rayt HS Bown MJ Lambert KV Fishwick NG McCarthy MJ London NJ Buttock claudication and erectile dysfunction after internal iliac artery embolization in patients prior to endovascular aortic aneurysm repair. Cardiovasc Intervent Radiol (2008) 31:728–34. 10.1007/s00270-008-9319-3 18338212

[17] Duff C Simmen HP Brunner U Bauer E Turina M. Gluteal necrosis after acute ischemia of the internal iliac arteries. Vasa (1990) 19:252–6. 2238821

[18] Karkos CD Wood A Bruce IA Karkos PD Baguneid MS Lambert ME. Erectile dysfunction after open versus angioplasty aortoiliac procedures: a questionnaire survey. Vasc Endovascular Surg (2004) 38:157–65. 10.1177/153857440403800208 15064847

[19] McDermott MM Ades P Guralnik JM Dyer A Ferrucci L Liu K Treadmill exercise and resistance training in patients with peripheral arterial disease with and without intermittent claudication: a randomized controlled trial. JAMA (2009) 301:165–74. 10.1001/jama.2008.962 19141764 PMC3268032

[20] Heidelbaugh JJ. Management of erectile dysfunction. Am Fam Physician (2010) 81:305–12. 20112889

[21] Dumville JC Lee AJ Smith FB Fowkes FG. The health-related quality of life of people with peripheral arterial disease in the community: the Edinburgh artery study. Br J Gen Pract (2004) 54:826–31. 15527608 PMC1324915

[22] Rose GA. The diagnosis of ischaemic heart pain and intermittent claudication in field surveys. Bull World Health Organ (1962) 27:645–58. 13974778 PMC2555832

[23] Gernigon M Le Faucheur A Noury-Desvaux B Mahe G Abraham P. Applicability of global positioning system for the assessment of walking ability in patients with arterial claudication. J Vasc Surg (2014) 60(973–81):e1. 10.1016/j.jvs.2014.04.053 24930016

[24] McDermott MM Liu K Guralnik JM Martin GJ Criqui MH Greenland P. Measurement of walking endurance and walking velocity with questionnaire: validation of the walking impairment questionnaire in men and women with peripheral arterial disease. J Vasc Surg (1998) 28:1072–81. 10.1016/S0741-5214(98)70034-5 9845659

[25] Le Faucheur A Abraham P Jaquinandi V Bouye P Saumet JL Noury-Desvaux B. Measurement of walking distance and speed in patients with peripheral arterial disease: a novel method using a global positioning system. Circulation (2008) 117:897–904. 10.1161/CIRCULATIONAHA.107.725994 18250268

[26] Leng GC Fowkes FG. The Edinburgh claudication questionnaire: an improved version of the WHO/rose questionnaire for use in epidemiological surveys. J Clin Epidemiol (1992) 45:1101–9. 10.1016/0895-4356(92)90150-L 1474406

[27] McDermott MM Greenland P Liu K Guralnik JM Criqui MH Dolan NC Leg symptoms in peripheral arterial disease: associated clinical characteristics and functional impairment. JAMA (2001) 286:1599–606. 10.1001/jama.286.13.1599 11585483

[28] Criqui MH Denenberg JO Bird CE Fronek A Klauber MR Langer RD. The correlation between symptoms and non-invasive test results in patients referred for peripheral arterial disease testing. Vasc Med (1996) 1:65–71. 9546918 10.1177/1358863X9600100112

[29] Inuzuka K Unno N Mitsuoka H Yamamoto N Ishimaru K Sagara D Intraoperative monitoring of penile and buttock blood flow during endovascular abdominal aortic aneurysm repair. Eur J Vasc Endovasc Surg (2006) 31:359–65. 10.1016/j.ejvs.2005.09.019 16364666

[30] Mahe G Leftheriotis G Picquet J Jaquinandi V Saumet JL Abraham P. A normal penile pressure cannot rule out the presence of lesions on the arteries supplying the hypogastric circulation in patients with arterial claudication. Vasc Med (2009) 14:331–8. 10.1177/1358863X09106173 19808718

[31] Currie IC Jones AJ Wakeley CJ Tennant WG Wilson YG Baird RN Non-invasive aortoiliac assessment. Eur J Vasc Endovasc Surg (1995) 9:24–8. 10.1016/S1078-5884(05)80220-5 7664007

[32] Bruninx G Salame H Wery D Delcour C. [Doppler study of gluteal arteries. A useful tool for excluding gluteal arterial pathology snd an important adjunct to lower limb Doppler studies]. J Mal Vasc (2002) 27:12–7. 12070836

[33] Hassen-Khodja R Batt M Michetti C Le Bas P. Radiologic anatomy of the anastomotic systems of the internal iliac artery. Surg Radiol Anat (1987) 9:135–40. 10.1007/BF02086598 3120332

[34] Klein AJ Feldman DN Aronow HD Gray BH Gupta K Gigliotti OS SCAI expert consensus statement for aorto-iliac arterial intervention appropriate use. Catheter Cardiovasc Interv (2014) 84:520–8. 10.1002/ccd.25505 24740523

[35] Tetteroo E van der Graaf Y Bosch JL van Engelen AD Hunink MG Eikelboom BC Randomised comparison of primary stent placement versus primary angioplasty followed by selective stent placement in patients with iliac-artery occlusive disease. Dutch iliac stent trial study group. Lancet (1998) 351:1153–9. 10.1016/S0140-6736(97)09508-1 9643685

[36] Hioki H Miyashita Y Miura T Ebisawa S Motoki H Izawa A Diagnostic value of peripheral fractional flow reserve in isolated iliac artery stenosis: a comparison with the post-exercise ankle-brachial index. J Endovasc Ther (2014) 21:625–32. 10.1583/14-4734MR.1 25290788

[37] Jaquinandi V Kaladji A Lederlin M Mahe G Re: “diagnostic value of peripheral fractional flow reserve in isolated iliac artery stenosis: a comparison with the post-exercise ankle-brachial index”. J Endovasc Ther (2015) 22:272–4. 10.1177/1526602815576093 25809375

[38] Tonino PA De Bruyne B Pijls NH Siebert U Ikeno F van’ t Veer M Fractional flow reserve versus angiography for guiding percutaneous coronary intervention. N Engl J Med (2009) 360:213–24. 10.1056/NEJMoa0807611 19144937

[39] Sugano N Inoue Y Iwai T. Evaluation of buttock claudication with hypogastric artery stump pressure measurement and near infrared spectroscopy after abdominal aortic aneurysm repair. Eur J Vasc Endovasc Surg (2003) 26:45–51. 10.1053/ejvs.2002.1870 12819647

[40] Mahe G Kalra M Abraham P Liedl DA Wennberg PW. Application of exercise transcutaneous oxygen pressure measurements for detection of proximal lower extremity arterial disease: a case report. Vasc Med (2015) 20:251–5. 10.1177/1358863X14567030 25750011

[41] Bouye P Jacquinandi V Picquet J Thouveny F Liagre J Leftheriotis G Near-infrared spectroscopy and transcutaneous oxygen pressure during exercise to detect arterial ischemia at the buttock level: comparison with arteriography. J Vasc Surg (2005) 41:994–9. 10.1016/j.jvs.2005.03.020 15944599

[42] Comerota AJ Throm RC Kelly P Jaff M. Tissue (muscle) oxygen saturation (StO2): a new measure of symptomatic lower-extremity arterial disease. J Vasc Surg (2003) 38:724–9. 10.1016/S0741-5214(03)01032-2 14560221

[43] Ubbink DT Koopman B. Near-infrared spectroscopy in the routine diagnostic work-up of patients with leg ischaemia. Eur J Vasc Endovasc Surg (2006) 31:394–400. 10.1016/j.ejvs.2005.10.025 16359878

[44] Jonason T Bergstrom R. Cessation of smoking in patients with intermittent claudication. Effects on the risk of peripheral vascular complications, myocardial infarction and mortality. Acta Med Scand (1987) 221:253–60. 10.1111/j.0954-6820.1987.tb00891.x 3591463

[45] WHO. Recommendations for Prevention of Cardiovascular Disease: Guidelines for Assessment and Management for Cardiovascular Risk. Geneva: World health organization (2007).

[46] Carsin-Mahe M Abraham P Le Faucheur A Leftheriotis G Mahe G Simple routine assessment of dietary pattern in patients with peripheral artery disease. J Vasc Surg (2012) 56:281–2. 10.1016/j.jvs.2012.02.059 22749274

[47] Gardner AW Parker DE Montgomery PS Scott KJ Blevins SM Efficacy of quantified home-based exercise and supervised exercise in patients with intermittent claudication: a randomized controlled trial. Circulation (2011) 123(5):491–8. 10.1161/CIRCULATIONAHA.110.963066 21262997 PMC3154843

[48] Antithrombotic Trialists Collaboration. Collaborative meta-analysis of randomised trials of antiplatelet therapy for prevention of death, myocardial infarction, and stroke in high risk patients. BMJ (2002) 324:71–86. 10.1136/bmj.324.7329.71 11786451 PMC64503

[49] CAPRIE Steering Committee. A randomised, blinded, trial of clopidogrel versus aspirin in patients at risk of ischaemic events (CAPRIE). CAPRIE steering committee. Lancet (1996) 348:1329–39. 10.1016/S0140-6736(96)09457-3 8918275

[50] Stone NJ Robinson JG Lichtenstein AH Bairey Merz CN Blum CB Eckel RH 2013 ACC/AHA guideline on the treatment of blood cholesterol to reduce atherosclerotic cardiovascular risk in adults: a report of the American college of cardiology/American heart association task force on practice guidelines. Circulation (2014) 129:S1–45. 10.1161/01.cir.0000437738.63853.7a 24222016

[51] Heart Protection Study Collaborative Group. Randomized trial of the effects of cholesterol-lowering with simvastatin on peripheral vascular and other major vascular outcomes in 20,536 people with peripheral arterial disease and other high-risk conditions. J Vasc Surg (2007) 45:645–54. 10.1016/j.jvs.2006.12.054 17398372

[52] Yusuf S Sleight P Pogue J Bosch J Davies R Dagenais G. Effects of an angiotensin-converting-enzyme inhibitor, ramipril, on cardiovascular events in high-risk patients. The heart outcomes prevention evaluation study investigators. N Engl J Med (2000) 342:145–53. 10.1056/NEJM200001203420301 10639539

[53] Ahimastos AA Walker PJ Askew C Leicht A Pappas E Blombery P Effect of ramipril on walking times and quality of life among patients with peripheral artery disease and intermittent claudication: a randomized controlled trial. JAMA (2013) 309:453–60. 10.1001/jama.2012.216237 23385271

[54] Thompson K Cook P Dilley R Saeed M Knowles H Terramani T Internal iliac artery angioplasty and stenting: an underutilized therapy. Ann Vasc Surg (2010) 24:23–7. 10.1016/j.avsg.2009.05.005 19631502

[55] Donas KP Schwindt A Pitoulias GA Schonefeld T Basner C Torsello G. Endovascular treatment of internal iliac artery obstructive disease. J Vasc Surg (2009) 49:1447–51. 10.1016/j.jvs.2009.02.207 19497505

[56] Picquet J Miot S Abraham P Venara A Papon X Fournier HD Crossed retroperitoneal approach to the internal iliac artery: a preliminary anatomical study. Surg Radiol Anat (2006) 28:180–4. 10.1007/s00276-005-0066-8 16341823

[57] Prince JF Smits ML van Herwaarden JA Arntz MJ Vonken EJ van den Bosch MA Endovascular treatment of internal iliac artery stenosis in patients with buttock claudication. PLoS One (2013) 8:e73331. 10.1371/journal.pone.0073331 23951349 PMC3738523

[58] Huetink K Steijling JJ Mali WP. Endovascular treatment of the internal iliac artery in peripheral arterial disease. Cardiovasc Intervent Radiol (2008) 31:391–3. 10.1007/s00270-006-0106-8 17610115

[59] Adlakha S Burket M Cooper C. Percutaneous intervention for chronic total occlusion of the internal iliac artery for unrelenting buttock claudication. Catheter Cardiovasc Interv (2009) 74:257–9. 10.1002/ccd.21966 19213070

[60] Morse SS Cambria R Strauss EB Kim B Sniderman KW. Transluminal angioplasty of the hypogastric artery for treatment of buttock claudication. Cardiovasc Intervent Radiol (1986) 9:136–8. 10.1007/BF02577922 2942256

[61] Smith G Train J Mitty H Jacobson J. Hip pain caused by buttock claudication. Relief of symptoms by transluminal angioplasty. Clin Orthop Relat Res (1992):176–80. 1395290

[62] Batt M Baque J Bouillanne PJ Hassen-Khodja R Haudebourg P Thevenin B. Percutaneous angioplasty of the superior gluteal artery for buttock claudication: a report of seven cases and literature review. J Vasc Surg (2006) 43:987–91. 10.1016/j.jvs.2006.01.024 16678694

[63] Paumier A Abraham P Mahe G Maugin E Enon B Leftheriotis G Functional outcome of hypogastric revascularisation for prevention of buttock claudication in patients with peripheral artery occlusive disease. Eur J Vasc Endovasc Surg (2010) 39:323–9. 10.1016/j.ejvs.2009.10.009 19910224

[64] Maugin E Abraham P Paumier A Mahe G Enon B Papon X Patency of direct revascularisation of the hypogastric arteries in patients with aortoiliac occlusive disease. Eur J Vasc Endovasc Surg (2011) 42:78–82. 10.1016/j.ejvs.2011.03.014 21531593

[65] Steg PG Bhatt DL Wilson PW D’Agostino R Sr. Ohman EM Rother J One-year cardiovascular event rates in outpatients with atherothrombosis. JAMA (2007) 297:1197–206. 10.1001/jama.297.11.1197 17374814

